# Gut microbiota dysbiosis with hepatitis B virus liver disease and association with immune response

**DOI:** 10.3389/fcimb.2023.1152987

**Published:** 2023-05-02

**Authors:** Fengna Yan, Qun Zhang, Ke Shi, Yi Zhang, Bingbing Zhu, Yufei Bi, Xianbo Wang

**Affiliations:** Center for Integrative Medicine, Beijing Ditan Hospital Capital Medical University, Beijing, China

**Keywords:** gut microbiome, liver disease, T cell immunosuppression, hepatocellular carcinoma, peripheral immune response

## Abstract

**Background and aims:**

Given hepatitis B virus (HBV)-related hepatocellular carcinoma (HBV-HCC) exhibits unique gut microbiota characteristics and a significant immunosuppressive tumor microenvironment. Thus, a better understanding of the correlation between gut microbiota and the immunosuppressive response may help predict occurrence and prognosis of HBV-HCC.

**Methods:**

Here, in a cohort of ninety adults (healthy control n=30, HBV-cirrhosis n=30, HBV-HCC n=30) with clinical data, fecal 16S rRNA gene sequencing, matched peripheral blood immune response with flow cytometry analysis. Correlation between the gut microbiome of significantly different in HBV-HCC patients and clinical parameters as well as the peripheral immune response was assessed.

**Results:**

We found that community structures and diversity of the gut microbiota in HBV-CLD patients become more unbalanced. Differential microbiota analysis that *p:Acidobacteriota, p:Proteobacteria, p:Campilobacterota, f:Streptococcaceae, g:Klebsiella* associated with inflammation were enriched. The beneficial bacteria of *f:Clostridia UCG−014, f:Oscillospiraceae, f:_Rikenellaceae, g:_Barnesiella, g:Prevotella, g:Agathobacter* were decreased. Functional analysis of gut microbiota revealed that lipopolysaccharide biosynthesis, lipid metabolism, butanoate metabolism were significantly elevated in HBV-CLD patients. Spearman’s correlation analysis showed that *Muribaculaceae, Akkermaniacaeae, [Eubacterium]_coprostanoligenes_group, RF39, Tannerellaceae* have positive correlation with CD3+T, CD4+T and CD8+T cell counts while negatively correlated with liver dysfunction. Furthermore, paired peripheral blood showed a decreased proportion of CD3+T, CD4+T and CD8+T cells, while an increased T (Treg) cells. The immunosuppressive response of programmed cell death 1 (PD-1), cytotoxic T-lymphocyte antigen 4 (CTLA-4), immune receptor tyrosine based inhibitor motor (ITIM) domain (TIGIT), T-cell immune domain, and multiple domain 3 (TIM-3) of CD8+T cells were higher in HBV-HCC patients. They were positively correlated with harmful bacteria, such as *Actinobaciota, Myxococota, Streptococcaceae* and *Eubacterium coprostanoligenes*.

**Conclusions:**

Our study indicated that gut beneficial bacteria, mainly *Firmicutes* and *Bacteroides* appeared dysbiosis in HBV-CLD patients. They have negative regulation of liver dysfunction and T cell immune response. It provides potential avenues for microbiome-based prevention and intervention for anti-tumor immune effects of HBV-CLD.

## 1 Introduction

Primary liver cancer is the sixth most common cancer in the world and the second most common cause of cancer deaths (Sung et al., 2021). Globally, hepatocellular carcinoma (HCC), which is the main type of liver cancer, accounts for approximately 75% of the total liver cancer cases (Petrick et al., 2020). HBV infection, which is the most significant risk factor associated with the development of HCC, accounts for approximately 50% of HBC-HCC cases (Akinyemiju et al., 2017). Therefore, the progression of chronic HBV infections to cirrhosis and liver cancer must be effectively monitored.

The etiology of HBV-CLD is complicated and involves viral, genetic and environmental factors (Chang et al., 2016). Currently, the gut microbiome is attracting considerable attention as a risk factor for liver disease progression (Ponziani et al., 2019). In adults, the abundance of *Escherichia coli* and *Megasphaera* is greatest in non-alcoholic steatohepatitis individuals compared to that in obese individuals. The abundance of *Proteobacteria* was greater in non alcoholic fatty liver disease (NAFLD) patients with fibrosis compared with that in nonfibrotics (Caussy et al., 2019). A recent study showed loss of *Akkermansia muciniphila* correlates with hepatic monocytic myeloid-derived suppressor cells abundance, its reintroduction restores intestinal barrier function and strongly reduces liver inflammation and fibrosis. Imbalanced gut microbiota fuels HCC development by shaping the hepatic inflammatory microenvironment (Schneider et al., 2022). Evidence increasingly indicates that the gut microbiome with its multiple metabolites is associated with not only the pathogenesis but also the treatment of HCC especially immune checkpoint inhibitor therapy (ICI) (Zhang et al., 2020; Mager Lukas et al., 2020). Studies have indicated that PD-1 treatment imparted significant benefits to HCC patients with a higher abundance of intestinal microorganisms, such as *Lachnospiraceae* bacterium GAM79 and *Alistipes* sp. Marseille P5997. The clinical progression-free survival (PFS) and overall survival (OS) periods of these patients were longer than those of patients with a low abundance of the above organisms. In addition, patients with longer PFS displayed a higher abundance of *Ruminococcus calidus* and *Erysipelotichacea* bacterium GAM147 (Peng et al., 2021). However, studies focused more on the changes of intestinal microecology in NAFLD and alcohol-related liver disease, there were few reports on the changes of intestinal microecology characteristics in HBV-CLD.

Increasing evidence suggests that gut microbes and metabolites participate in many aspects of immunity, including the development, differentiation, and function of immune cells in the tumor microenvironment. According to reports, microbiota produce short chain fatty acid (SCFA) derivatives, such as butyrates, which promote cell metabolism, activate CD8+ T cell memory potential, stimulate antigens necessary for the rapid generation of antitumor effects, and give rise to microorganism-related metabolites of CD8+ T cells, all of which play a role in the survival of long-term memory cells (Bachem et al., 2019). Studies have demonstrated that gut microbes may strengthen the tumor immunosuppressive environment of NAFLD-HCC patients by increasing the number of regulatory T cells (Treg) and reducing that of CD8+ T cells (Behary et al., 2021). In conclusion, understanding gut microbes as well as their metabolic characteristics that affect HBV-HCC may provide a unique opportunity to develop prognostic markers and new treatments. Therefore, we compared the dysbiosis of gut microbes in HBV-LC and HBV-HCC patients with healthy individuals. Then, we analyzed gut microbes impact on the peripheral immune response and multiple clinical indicators in order to identify potential gut microbiota that significantly regulate anti-tumor immune effects. It may help improve the immune regulation of HBV- HCC.

## 2 Materials and methods

### 2.1 The study population

The HBV-CLD patients were recruited from the Center for Integrative Medicine, Beijing Ditan Hospital Capital Medical University. Three groups were consecutively recruited as follows: (i) participants with HBV-HCC (n=30); (ii) participants with HBV-cirrhosis (n=30); and (iii) healthy controls, consisting of healthy volunteers who visited our hospital (n=30). In order to ensure the consistency of microbiota among all participants were required to complete a questionnaire on their eating habits during the preceding six months, including information regarding the types of carbohydrates, fatty foods, vegetables, and fruits consumed. The HBV-HCC inclusion criteria were as follows: (i) patients diagnosed with primary liver cancer; (ii) aged 18-75 years; (iii) patients with BCLC C and D stage; (iv) patients had a history of chronic hepatitis B (HBsAg) positive > 6 months; (v) patients were not treated with drugs to regulate gut microbes during the past 1 month. Exclusion criteria were as follows: (i) patients with cholangiocarcinoma; (ii) patients with metastatic liver cancer; (iii) patients comorbid with other types of tumor; (iv) patients who were lost to follow-up; (v) patients with incomplete clinical data. Health controls inclusion criteria: (i) Health controls with the World Health Organization’s definition of health (i.e., in good physical, mental, and social conditions); (ii) Health controls have no abnormalities in liver function testing; (iii) Health controls have no history of alcohol consumption. Clinical and demographic characteristics of all patients are summarized (**Table 1**). The study was approved by the ethics committee of Beijing Ditan Hospital, Capital Medical University and was conducted in accordance with the standards of the Declaration of Helsinki.

**Table 1:** Demographic and clinical characteristics of patients with HBV-LC, HBV-HCC.

	Total(n=59)	LC n=30 (%)	HCC n=29 (%)	p
Patients background				
Age (mean ± SD)	54.644 ± 9.950	51.333 ± 9.789	58.069 ± 8.901	0.009
Gender(male/female) n(%)	48/11 (18.6/81.4)	25/5(83.3/16.7)	23/6 (79.3%/20.7)	0.692
Family history of LC n(%)	34 (57.63)/25(42.37)	20 (66.67)/0(33.33)	14 (48.28)/15(51.72)	0.153
Smoke (Yes/No) n(%)	35 (59.32)/24(40.68)	16 (53.33)/14(46.77)	19 (65.52)/10(34.48)	0.341
Drink (Yes/No)	34 (57.63)/25 (42.37)	14 (46.67)/16(53.33)	20 (68.97)/9(31.03)	0.083
Hypertension, n(%)	40 (67.80)/19 (32.20)	21 (70.00)/9(30.00)	19 (65.52)/10(34.48)	0.713
Diabetes, n(%)	41 (69.49)/18 (30.51)	22 (73.33)/8(26.67)	19 (65.52)/10(34.48)	0.514
Hypersplenism, n(%)	18 (30.51)/41 (69.49)	3 (10.00)/27(90.00)	15 (51.72)/14(48.28)	<0.001
HBV-related indicators				
HBV DNA(Positive/Negative)	41 (69.49)/18 (30.51)	24 (80.00)/6(20.00)	17 (58.62)/12(41.38)	0.075
HBeAg (IU/ml)	0.40 (0.32,1.57)	0.37 (0.32,1.67)	0.41 (0.33,1.46)	0.379
Laboratory data				
lymphocyte(10^9^/L), median	0.81 (0.50,1.49)	0.81 (0.51,1.49)	0.78 (0.48,1.44)	0.716
Neutrophils (10^9^/L), median	2.43 (1.58,3.42)	2.04 (1.16,2.72)	2.96 (2.35,3.73)	0.001
Leukocyte (10^9^/L), median	4.02 (2.92,5.87)	3.39 (2.22,4.22)	4.41 (3.38,5.97)	0.036
NLR	2.56 (1.70,4.46)	1.93 (1.408,3.33)	3.21 (2.38,5.59)	0.004
PLT(10^9^/L)	93 (62,128)	86 (45,115)	102 (78,144)	0.055
AST(U/L)	37.30 (25.90,67.20)	32.20 (25.80,45.40)	39.40 (27.90,122.70)	0.092
ALT(U/L)	26.3 (19.6,43.2)	26.0 (18.6,37.5)	30.1 (21.7,52.2)	0.252
ALB(g/L)	33.69 ± 5.56	32.39 ± 5.51	35.05 ± 5.29	0.068
TBIL (umol/L)	22.2 (13.2,43.7)	19.1 (13.7,42.7)	24.1 (13.2,47.8)	0.467
GGT(U/L)	125.34 ± 210.08	55.80 ± 85.02	199.84 ± 270.08	0.012
LDH(U/L)	226.00 ± 91.08	204.99 ± 76.86	247.77 ± 99.19	0.079
TG(mmol/L)	0.89 ± 0.55	0.85 ± 0.54	0.93 ± 0.55	0.567
CRP(mg/L)	10.41 ± 20.27	4.17 ± 7.42	17.35 ± 26.74	0.021
CREA(uml/L)	65.0 (55.4,73.0)	64.7 (54.0,68.8)	65.5 (57.2,78.8)	0.564
eGFR(ml/min)	104.9 (94.8,112.6)	107.8 (98.8,116.2)	98.6 (89.7,110.1)	0.080
Prothrombin times(s)	13.7 (12.6,15.3)	14.0 (2.9,16.4)	13.7 (12.6,14.2)	0.145
Tumor-related indicators				
AFP (ng/ml)	5.03 (2.27,46.08)	2.48 (2.04,5.38)	46.08 (4.89,459.73)	<0.001
Child grade A	21 (35.593)	8 (26.667)	13 (44.828)	0.292
B	28 (47.458)	17 (56.667)	11 (37.931)	
C	10 (16.949)	5 (16.667)	5 (17.241)	

Values are represented as mean ( ± SD) or %. P values. P < 0.05 comparison between HCC considered statistically significant. SD, standard deviation; NLR, Neutrophil‐lymphocyte ratio; ALT, alanine aminotransferase; AST, aspartate aminotransferase; γ-GGT, γ-glutamyl transferase; AFP, alpha-fetoprotein; CRP, C reactive protein; ALB albumin; HBeAg, hepatitis B e antigen; TBIL, total bilirubin; HBsAg, hepatitis B s antigen; CREA, creatinine; LDH, lactate dehydrogenase; TG, triglyceride.

### 2.2 Laboratory examinations

We collected fecal samples from all participants and additionally collected paired peripheral blood from HBV-CLD patients on the day of enrollment. Peripheral blood was storage at 4°C and used to separate mononuclear cells (PBMCs). Fresh stool samples were placed in sterile plastic tubes and stored within 15 min at −80°C, until needed. Total bacterial DNA was isolated using a MagPure Soil DNA KF Kit (Magen, Germany) according to the manufacturer’s instructions. Next, 5 mL of each patient’s whole blood was centrifuged at 700×*g* for 10 min to separate plasma, treated with a similar volume of PBS, gently mixed, spread on 3 mL Ficoll-Paque density gradient media (Merck, # 26873-85-8), and centrifuged at 700×*g* for 20 min, following which PBMCs were harvested for mesosphere experiments.

### 2.3 DNA extraction and PCR amplification

Total genomic DNA was extracted using a DNA Extraction Kit (Magen, #D6356-02) following the manufacturer’s instructions. The quantity and quality of DNA was verified using NanoDrop and agarose gel analysis. Extracted DNA was diluted to a concentration of 1 ng/μl and stored at –20°C until needed for further processing. The diluted DNA was used as a template for PCR amplification of bacterial 16S rRNA genes with the barcoded primers and Takara Ex Taq (Takara, #RR001Q). For the purpose of analyzing bacterial diversity, the V3-V4 variable regions of 16S rRNA genes were amplified *via* the universal primers 343F and 798R, whereas the V4-V5 variable regions were amplified using 515F and 907R. To analyze the diversity of eukaryota, variable regions of 18S rRNA genes were amplified using the universal primers, 817F and 1196R. The ITS I variable regions were amplified using the universal primers, ITS1F and ITS2, for fungal diversity analysis. Amplicon quality was visualized using gel electrophoresis, purified with AMPure XP beads (Agencourt), and amplified for another round of PCR. After being purified with the AMPure XP beads again, the final amplicon was quantified using a Qubit dsDNA assay kit (yeasen, #12642ES76). Equal amounts of purified amplicon were pooled for subsequent sequencing.

### 2.4 Flow cytometry of human PBMCs

Lymphocytes were transferred into FACS tubes and stained with fluorescence-conjugated monoclonal antibodies as follows; anti-human BV785-conjugated anti-CD3 (1:100; Biolegend, # 344842), APC-Fir750-conjugated anti-CD4 (1:100; BD, # 563800), BV510-conjugated anti-CD8 (1:100; Biolegend, #344732), PE-CF594-conjugated anti-CD25 (1:100; Biolegend, #356126), PE-conjugated anti-CD127 (1:100; Biosciences, #557938), AF700-conjugated anti-CD45RA (1:100; Biolegend, #304120), BV421-conjugated anti-CCR7 (1:25; Biolegend, # 353208), FITC-conjugated anti-CD38 (1:100; eBioscience, #11-0388-42), BV605-conjugated anti-HLA-DR (1:100; Biosciences, #564017), BV711-conjugated anti-PD-1 (1:100; Biosciences, #564017), PE-CY7-conjugated anti-TIGIT (1:100; eBioscience, #25-9500-42), BV650-conjugated anti-Tim-3 (1:100; Biosciences, #565564), APC-conjugated anti-CTLA4 (1:100; Biosciences, #349908), and corresponding isotype controls. Data were acquired using a LSR Fortessa flow cytometer and analyzed with FlowJo software (Tree Star).

### 2.5 Bioinformatic and statistical analysis

Raw sequencing data were in FASTQ format. Paired-end reads were preprocessed using cutadapt software to detect and remove the adapter. Then paired-end reads with low quality sequences were filtered, trimmed, denoised, and merged, following which chimera reads were detected and removed using DADA2 with the default parameters of QIIME2 (2020.11). Finally, a software output indicating the representative reads and the amplicon sequence variant (ASV) abundance table was obtained. Reads representative of individual ASVs were selected using the QIIME2 package. All representative reads were annotated and blasted against the Silva database, Version 138 (or Unite) (16s/18s/ITS rDNA) using a q2-feature-classifier with the default parameters.

Next, α-diversity which compares the bacterial abundance and diversity in samples was evaluated using the relative abundance distribution of OTUs in a single sample, and the QIME calculation which estimates the four indexes, Chao1, Simpson, Shannon and ACE. In addition, β-diversity that assesses the structure and distribution of microbial genetic communities in the sample was evaluated using the weighted UniFrac distance metric following which MEGAN (Version 4.70.4) software was utilized to visualize the classification composition and abundance.

Linear discriminant analysis Effect size (LEfSe) as well as Kruskal–Wallis and Bonferroni post‐analyses were used to identify groups showing significantly different microbiomes. Functional genes were predicted using PICRUSt2 and KEGG databases. All *p* values were corrected using Benjamini–Hochberg multiple test correction. Statistical significance was set at *p <*0.05.

Baseline statistics and polychromatic flow staining results of clinical patients were analyzed using GraphPad 5.0 and SPSS version 19.0. Consistent quantitative data were expressed as mean ± standard deviation (SD) and analyzed using the t-test. Data that were not normally distributed were expressed as the median of the quartile range and analyzed using the Mann–Whitney U test. Pearson’s and Spearman’s correlation coefficients were used to evaluate the correlation between normal and non-normal data, respectively. Again, statistical significance was set at *p*<0.05.

## 3 Results

### 3.1 Baseline characteristics of the study cohort

Based on inclusion and exclusion criteria, 90 participants were selected for this study and categorized into three groups: HBV-HCC (n=30), HBV-LC (n=30), and healthy controls (n=30). The two latter groups are collectively referred to as the HBV-CLD group. Clinical data, including background, HBV related indicators, laboratory data and tumor related indicators were collected from the HBV-CLD group on the day of inclusion. The results indicated that there was no difference between the smoking habits, drinking history, hypertension, diabetes, HBV family history, HBV virus characteristics, liver function, renal function of the group members. However, the levels of inflammation indicators, such as hypersplenism, neutrophils, neutrophil to lymphocyte (NLR) ratios and C reactive proteins (CRP) of the HBV-HCC group were higher compared with HBV-LC group (Table 1).

### 3.2 16S ribosomal DNA gene ASV abundance statistics

**Figure 1:**
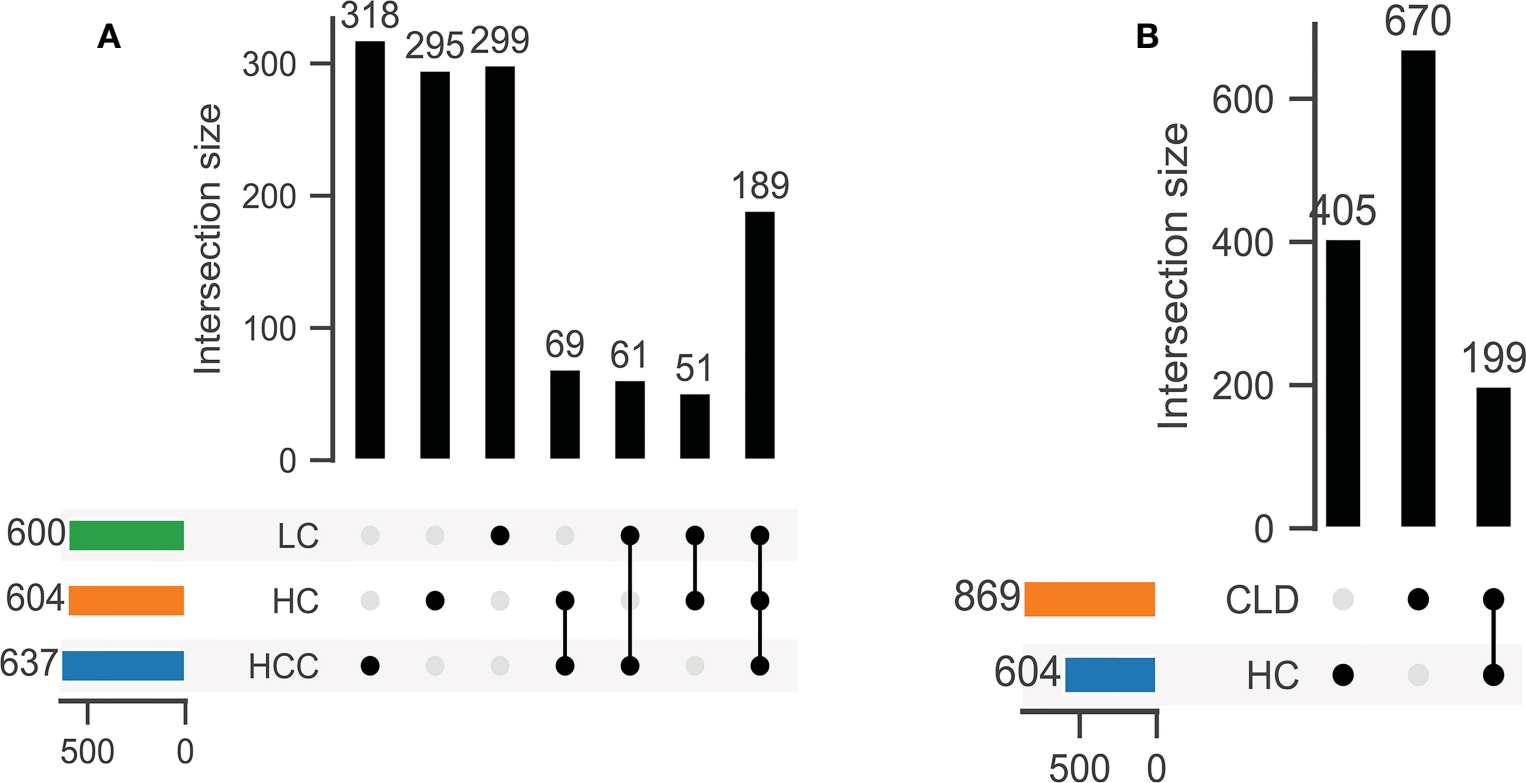
The number of common OTUs was significantly different between healthy control (HC) and CLD (HBV-LC, HBV-HCC) groups. **(A)** healthy control, HBV-LC, and HBV-HCC group; **(B)** healthy control and CLD group.

DNA extracted from 90 stool samples, which were obtained from the three groups, were sequenced to generate a V3-V4 16S rDNA gene spectrum. The number of ASVs in each sample was between 76 and 346 (**Figure 1A**). The number of common and unique OTUs among healthy controls, HBV-LC, and HBV-HCC patients were 405, 670, and 199, respectively (**Figure 1B**).

### 3.3 Phylum and family level community structures of the gut microbiota of healthy controls, HBV-LC, and HBV-HCC patients

**Figure 2:**
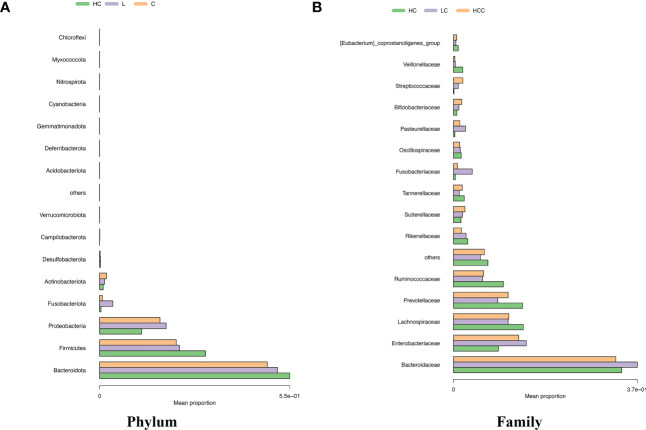
Distinct fecal microbiota profiles the top 15 of participants in the HC, HBV-LC, and HBV-HCC groups. Percentage of gut microbiota at the phylum **(A)** and family **(B)** levels.

**Figure 3:**
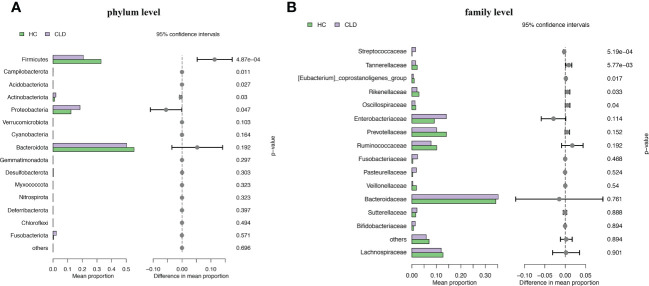
Microbiome composition at phylum level **(A)** and family level **(B)** showing top 15 most abundant families in HC and HBV-CLD fecal samples.

We analyzed the composition of gut microbiota in each group. Studies have indicated that the phylum level abundance of *Bacteroides* and *Firmicutes* in patients with HBV-LC and HCC gradually decreased, and *Proteobateria* and *Actinobaciota* increased with disease progression (**Figure 2A**). The family level abundance of beneficial bacteria, such as *Bacteroidaceae*, *Lachnospiraceae*, *Prevotelaceae*, *Ruminocochaceae*, *Rikenellaceae, Oscillospiraceae* of HBV-LC and HBV-HCC groups were found to be decreased (**Figure 2B**), whereas the known aggravators of intestinal inflammation, such as *Enterobacteriaceae, Suttrellaceae*, and *Streptocochaceae*, in HBV-LC and HBV-HCC patients was increased compared with healthy control. Furthermore, we classified HBV-LC and HBV-HCC patients into the HBV-CLD group and analyzed the microbial community structure. We observed that phylum level of *Bacteroidota* and *Firmicutes* was significantly reduced, while *Proteobateria*, *Actinobaciota*, *Fusobasteriota* was significantly increased (**Figure 3A**). Moreover, the family level of microbiota such as *Bacteroidaceae, Enterobacteriaceae, Sutterellaceae, Bifidobacteriaceae, Streptococcaee*, *Pasteurellacaee*, which are involved in intestinal inflammatory reactions of HBV-CLD patients was significantly increased. However, the abundance of *Lachnospiraceae, Ruminococcaceae, Prevotellaceae, Oscillospiraceae*, and *Rikenellaceae*, belonging to *Firmicutes* and *Bacteroidota of* HBV-CLD patients was significantly decreased. These gut microbiota are mainly involved in the synthesis and metabolism of short-chain fatty acids (**Figure 3B**).

### 3.4 The relationship between gut microbiota diversity and disease progression

**Figure 4:**
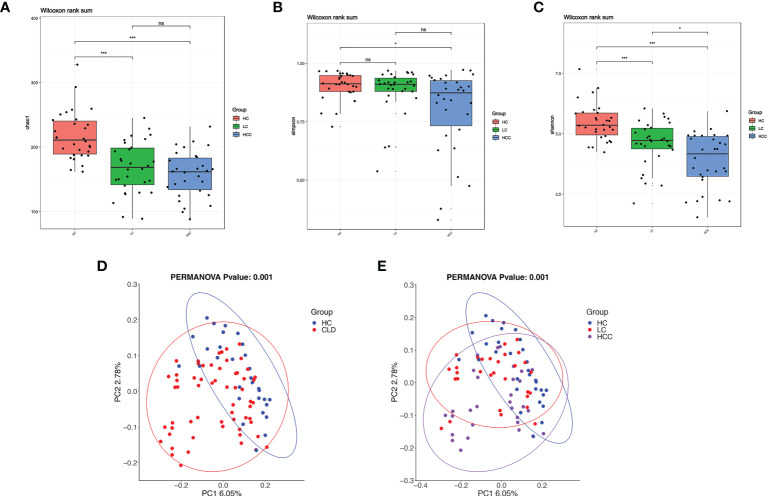
With the development of disease, the diversity of fecal microbiome gradually decreases. **(A–C)** Alpha-diversity based on observed number of species in HC controls, HBV-LC, and HBV-HCC fecal samples. Box plots indicate the median (middle line) and the 25^th^ and 75th percentiles (box). The figure shows the distribution of the diversity index in each group and indicates whether there is a significant difference in the diversity index between the three groups. **(D, E)** Beta-diversity using constraint analysis of principal components analysis (PCoA). Each sample is represented by a dot. Ellipses are added with function ‘stat_ellipse’ with a confidence level of 0.95. *P < 0.05, ****P < 0.0001; and ns indicates no significant difference. **(A–E)** sample size is healthy control, n = 30, HBV-LC, n = 30, HBV-HCC, n = 30 as biologically independent samples.

The α-diversity indices, Chao1 estimator, ACE estimator, Shannon index, Simpson index can be used to evaluate the abundance and diversity of microbial communities in intestinal fecal samples, while the microbial community structure among different groups of fecal samples can be analyzed using β-diversity. Compared with healthy controls, the α-diversity indices, including Chao, Shannon’s, Simpson index, decreased and it was significantly related to the aggravation of the disease course (**Figures 4A–C**). The α-diversity indicators, such as observed_ species, PD_ whole_ Tree, ACE estimator did not differ between groups (**Supplementary Figure 1**). The β-diversity as determined by the application of constrained principal components analysis (CPCA) to principal components analysis (PCA) was significantly different between three group (*p*=0.004) (**Figure 4E**). In addition, compared with the healthy control, the microbial diversity of samples in HBV-CLD group was consistent with the above results (**Figure 4D**).

**Figure 5:**
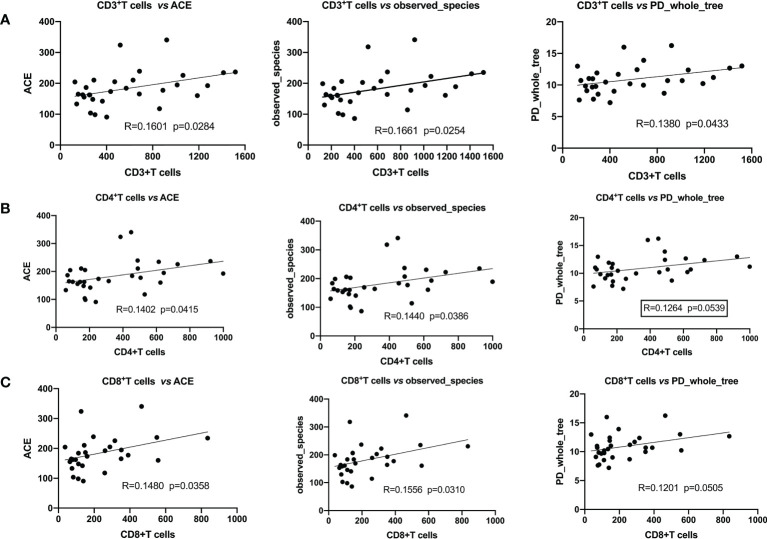
Pearson’s correlation indicated that α-diversity and immune cells were significantly positively correlated. **(A)** CD3+T cells with ACE, the observed_ species, PD_ whole_ Tree; **(B)** CD4+T cells with ACE, the observed_ species, PD_ whole_ Tree; **(C)** CD8+T cells with ACE, the observed_ species, PD_ whole_ Tree.

We analyzed the correlation between α-diversity of HBV-HCC patients and their immune cells. We found that α-diversity indices, such as observed_ species, PD_ whole_ Tree, ACE were significantly and positively correlated with counts of immune cells (CD3^+^, CD4^+^, CD8^+^T), indicating that intestinal microorganisms were closely associated with anti-tumor immune effects (**Figure 5**).

### 3.5 Differential abundance of bacterial taxa among healthy control, HBV-LC, and HBV-HCC groups

**Figure 6:**
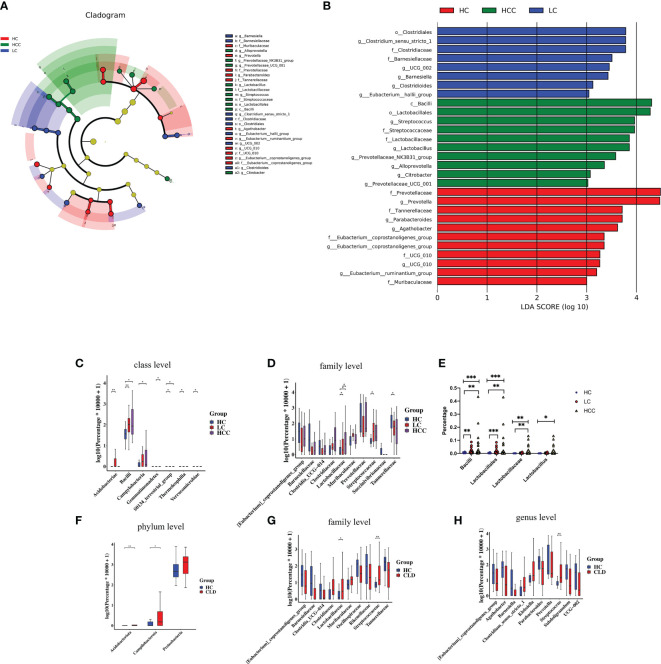
The abundance of beneficial bacteria was decreased, while that of harmful bacteria was increased in healthy controls as well as CLD (HBV-LC, HBV-HCC) patients. **(A)**: Histogram of LDA scores for differentially abundant taxa between HC, HBV-LC, and HBV-HCC groups; bar length indicates effect size associated with a taxon. Kruskal–Wallis threshold = 0.05, Wilcoxon test threshold = 0.05; (LDA>3, p<0.05). **(B)**: Taxonomic Cladogram from LEfSe; red, blue, and red show taxa enriched in HC, HBV-LC, and HBV-HCC. Each node represents species classification at this level. The higher the species abundance, the larger the node. **(C, D)**: Microbiome composition at class and family levels illustrating most enriched species in the fecal samples of HBV-LC and HBV-HCC groups compared to that of HC. **(E)**: *Lactobacillus* level after extending the time of drug without oral regulation of intestinal flora ≥2 months. **(F–H)**: Microbiome composition at the phylum, family, and genus levels illustrating most enriched species in HBV-CLD compared to HC group fecal samples; *P < 0.05, **P < 0.01, ***P < 0.001.

We used linear discriminant analysis (LDA) effect size (LEfSe) modeling to reveal the composition of different species in the three groups. Our differential analysis identified that the healthy control group was enriched in 11 OTUs, the HBV-LC group was enriched in 8 OTUs, the HBV-HCC group was enriched in 10 OTUs (LDA>3, *p*<0.05). Notably, the taxa, *f: Prevotellaceae and g: Prevotella* showed the highest concentration among the HBV-HCC group, which was also enriched in the taxa, *c:Bacilli, o: Lactobacillales, g: Streptococcus*, and *f: Streptococcuscaeae* (LDA>4, *p*<0.05) (**Figure 6A**). The Wilcoxon rank sum test and Kruskal–Wallis rank sum test were used to analyze differential abundance at the class and family levels (Metastats analysis). We found that the HBV-HCC group was most enriched in *p:Acidobacteriota, p:Proteobacteria, p:Campilobacterota, f:Lactobacillaceae, f:Streptococcaceae, g:Klebsiella*, and *g:Streptococcus* (LDA>4, p<0.05), (**Figures 6B–D**). The abundance of beneficial bacteria such as, *f:Clostridia UCG−014, f:Oscillospiraceae, f:_Rikenellaceae, g:_Barnesiella, g:Prevotella*, and *g:Agathobacter* is reduced in HBV-CLD group (**Figures 6F–H**). Similar results were observed at the class and order level (**Supplementary Figure 2**). Interestingly, the abundance of *c:Bacilli, o:Lactobacillales*, *f:Lactobacillaceae* in the HCC group was significantly increased. Hence, we further divided the clinical medication histories of patients in the HBV-HCC group and adjusted the time of application of microbiota regulating drugs in the inclusion criteria to 2 months and obtained the same results (**Figure 6E**). Therefore, we focused on lactic acid metabolites *via* a follow-up study.

### 3.6 Functional analysis of gut microbiota *via* 16S rDNA sequencing

**Figure 7:**
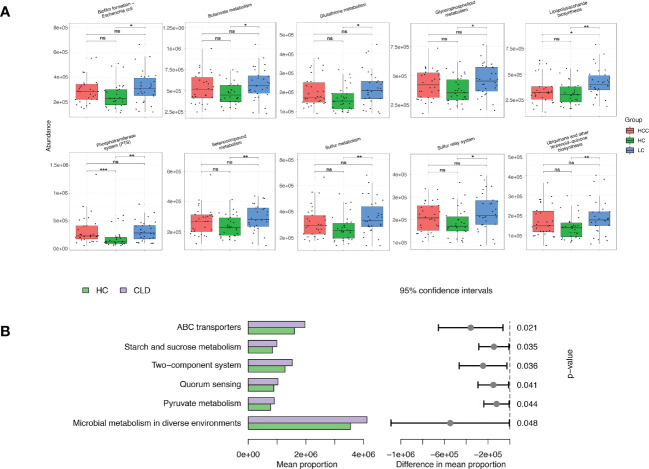
Functional analysis of three groups of intestinal microbiomes using 16S rDNA sequencing data indicating metabolic changes of energy substances. PICRUSt2 software was used to predict the composition of known microbial gene functions in different groups and count the functional differences between different samples with HC, HBV-LC, HBV-HCC **(A)** and HBV-CLD **(B)** groups. *P < 0.05, **P < 0.01, ***P < 0.001, ns, not significant.

We evaluated 16S rDNA data using PICRUSt2 (2.3.0b0) software and KEGG enrichment analysis to predict the functions of fecal microorganisms in three groups. The bacterial gene functional compositions of the fecal microorganisms in HBV-LC and HBV-HCC groups were significantly different from healthy control. The most abundant top 10 functions and signaling pathways were butanoate metabolism, glycerophospholipid metabolism, glutathione metabolism, lipopolysaccharide biosynthesis, sulfur metropolis, ubiquinone and other terpenoid-quinone biosynthesis, selenocompound metabolism, sulfur relay syster, biofilm forming Escherichia coli and the phosphotransferase system (PTS) (*p*<0.05) (**Figure 7A**).

The same calculation was used to analyze and predict the bacterial gene functions showing obvious differences between the healthy control and HBV-CLD patients. The most abundant bacterial genes were those associated with microbial metabolism in diffuse environments, pyruvate metabolism, quorum sensing, starch and sucrose metabolism, and ABC transporters (**Figure 7B**). Although the versatility of functional prediction based on 16 rRNA data is limited, it still reflects the metabolic processes that are active in HBV-CLD patients, mainly indicating that their pyruvate metabolism and carbohydrate metabolism are significantly different from healthy control. It may play an important role in the underlying mechanism of HBV-LC and HCC.

### 3.7 Correlations between the gut microbiota of HBV-HCC patients and clinical biochemical parameters

**Figure 8:**
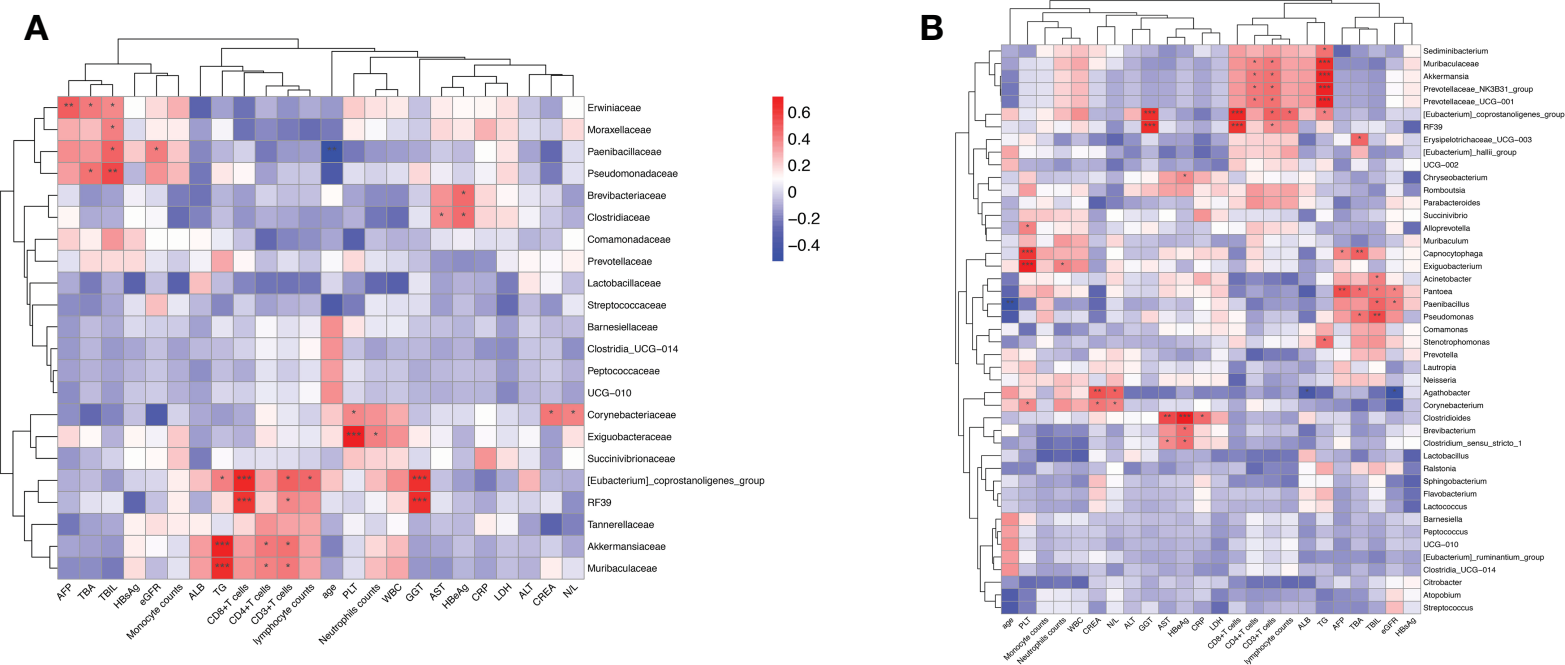
The intestinal microbiome of HBV-HCC patients at the family level is correlated with clinical liver function, lipid metabolism, and immune cell count. Heatmap of the correlation analysis between predominant bacterial families **(A)** and genera **(B)** and the clinical biochemical parameters of patients with HBV-HCC. Red color indicates a positive correlation while blue color indicates a negative correlation; *p<0.05; **p<0.01; ***p<0.001.

We utilized the stool and peripheral blood of HBV-HCC patients to observe the correlation between gut microbiota and clinical data. The clinical data of age, leukocyte count, alanine aminotransferase (ALT), aspartate aminotransferase (AST), total bilirubin (TBIL), γ-glutamyl transferase (GGT), AFP, Neutrophil‐lymphocyte ratio (NLR), HBsAg, HBeAg, lactic dehydrogenase (LDH), count of immune cells, CRP, and albumin (ALB) were collected. The Spearman correlations of the gut microbiome at the family and genus level of HBV-HCC with related variables are described (**Figure 8A**). Spearman’s correlation analysis shows that the significantly lessened bacteria of *Akkermaniacaee, Muribaculateae, (Eubacterium) Coprostanoligenes Group, RF39* in family level were positively correlated with the proportion of CD3+, CD4+ and CD8+ T cells and ALB (*p*<0.05). Moreover, they have negative correlation with indicators of AST, ALT, TBIL, LDH, CRP, AFP. Similarly, significantly reduced genus level of *Akkermansia, Prevotellaceae_*NK3B31_group*, Prevotellaceae_UCG-001, Eubacterium_coprostanoligenes_*group RF39*, Muribaculaceae* showed a positive correlation with count of CD3+, CD4+, CD8+ T cells (**Figure 8B**). The significantly increased bacteria of *Acinetobacter, Pantoea, Paenibacillus*, and *Pseudomonas* was positively correlated with the total bile acid (TBA) and triglyceride (TG), which were closely related to bile acid metabolism.

### 3.8 HBV-HCC showed stronger immunosuppressive response, closely related to gut microbiota

**Figure 9:**
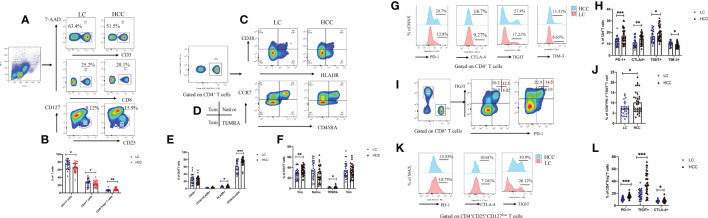
Compared with that of HBV-LC patients, the HBV-HCC patients had more severe peripheral immunosuppression phenotype. Fold change from baseline and representative flow cytometry plots of CD3+ T cells (CD3+), CD8+ T cells (CD3+CD8+), and Tregs (CD3+CD4+CD25hiCD127low) in response to HC, HBV-LC, and HBV-HCC. Data is represented as % of viable singlets for CD3+T cells, % of viable CD3+ lymphocytes for Tregs and CD8+ T cells, and % of viable CD4+, CD8+ T lymphocytes for *T*_N_, *T*_CM_, *T*_EM_, *T*_EMRA_ cells, HLA-DR+, CD38+, PD-1, CTLA-4, TIM-3, and TIGIT, normalized to baseline measurements. For panels **(A–L)** sample size is n=30 for HBV-LC, and n=30 for HBV-HCC (as biologically independent samples). Box plots indicate the median (middle line) and the 25th and 75th percentiles (box). *P* values are calculated using Tukey’s test for 2 group comparisons. Non-normally distributed data were expressed using the median with quartile ranges and analyzed *via* the Mann–Whitney U test; *P < 0.05, **P < 0.01, ***P < 0.001.

Many studies have shown that a variety of intestinal microorganisms and metabolites are able to positively regulate the anti-tumor immune effect (Macpherson et al., 2016; Nearing et al., 2022). We confirmed that the proportion of CD3+ and CD8+ T cells in the peripheral blood of HBV-HCC patients decreased significantly, while the Tregs increased compare with HBV-LC group (*p*<0.05) (**Figures 9A, B**). In addition, we determined the frequency of CD38^+^, HLA-DR^+^, central memory (*T_CM_*), and terminally differentiated effector (*T_EMRA_*) cells, which respectively represent the activation and memory differentiation phenotypes on CD4^+^ T cell population in HBV-HCC was significantly higher than HBV-LC patients (*p*<0.05) (**Figures 9C–F**). In addition, the percentage of PD-1, CTLA-4, TIGIT, and TIM-3 co-inhibitory receptors of CD4+, CD8+T cells and Treg cell has increased significantly (*p*<0.05) (**Figures 9G–L**). Next, we further analyzed the gut microbiota composition related to significantly changed immunosuppressive phenotypes.

**Figure 10:**
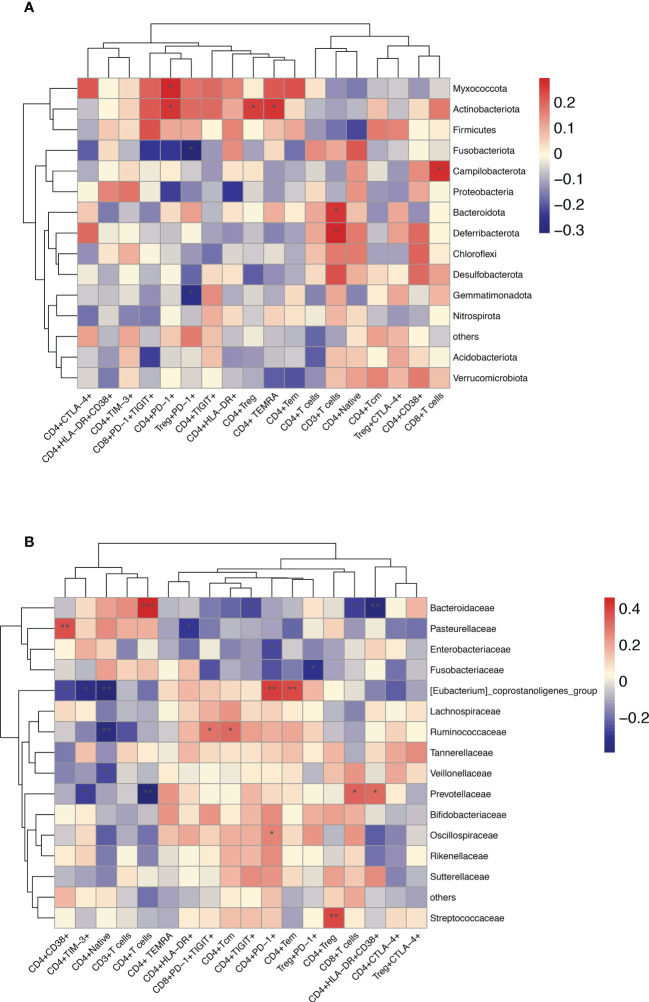
Enriched species in the microbiome of participants with HBV-HCC were correlated with the peripheral immune response. Heatmap of Spearman’s rank correlation of enriched species in the microbiome and T cell immune responses at the phylum **(A)** and family **(B)** level with HBV-HCC group. Color legend represents correlation coefficient; “+” denotes significant correlation (P < 0.05) after Benjamini–Hochberg correction. *P < 0.05, **P < 0.01, ***P < 0.001.

Furthermore, in HBV-HCC patients, it was observed that *Bacteroidota, Deferribacterota* and CD3+T cells, *Campilobacterota*, *Akkermaniacaeae* and CD8+T cells, *Actinobaciota* and Treg cells, *Actinobaciota, Myxococota* and CD4+PD-1+T cells, *Actinobacteriota* and CD4+T*_EMRA_* cells at phylum level were positively correlated, while *Gemmatimonadota* and *Fusobasteriota* were negatively correlated with the percentage of Treg+PD-1+T cells (**Figure 10A**). At the family level, *Bacteroidaceae* and CD4+T cells, *Prevotelaceae* and CD8+T cells, *Streptococcaceae* and Tregs cells, *[Eubacterium]_coprostanoligenes_Group* and CD4+PD-1+T cells, *Ruminococcuscaeae* and CD4+*T_CM_* cells, *Pasteurellacaeae* and CD4+CD38+T cells, *Prevotellacaeae* and CD4+HLA-DR+CD38+T cells, as well as *[Eubacterium]_ coprostanoligenes_ Group*_ and CD4+PD-1+T cells have significantly positively correlation, *Fusobacteriacaee* and Tregs+PD-1+T cells, *Ruminococcaceae* and naïve CD4+T cells (*T_N_*), *[Eubacterium]_ coprostanoligenes_ group* and CD4+TIM-3+T cells, *Bacteroidacea* and CD4+HLA-DR+CD38+T cells were negatively correlated (p<0.05) (**Figure 10B**). Flow cytometry results showed that HBV-HCC showed a stronger immunosuppressive response, which in turn was linearly related to gut microbiota. Considered together, these results may provide an important indicator for tumor immunity researches.

## 4 Discussion

According to mainstream research, the gut microbiota participates in the pathophysiological process of tumors *via* intermediate metabolism, bile secretion, maintaining blood sterility, serum homeostasis, exogenous detoxification, and immune activity conducted through the gut-liver axis (Matson et al., 2018; Zheng et al., 2019). Intestinal microbiota and its metabolites reportedly promote the occurrence and progression of HCC, but there are few reports on the relevance of gut microbiota with HBV liver diseases immunosuppressive phenotype (Wan and El-Nezami, 2018; Zeng et al., 2020). Our studies utilized the 16S rRNA gene sequencing database to determine the structural and functional changes of microbiomes that distinguish between healthy people and HBV-LC and HBV-HCC patients. Moreover, the paired anti-tumor immune response phenotypes of HBV-LC and HBV-HCC patients was detected. Then, we used correlation analysis to determine the association between the significantly different microbiomes and clinical as well as immune indicators of HBV-HCC patients with the expectation that such findings would be clinically significant.

In our study, Chao1, goods_ coverage, Shannon, observed_ species, Simpson, PD_ whole_ Tree, and ACE diversity indices which reflect α-diversity were significantly reduced in HBV-HCC patients. However, a previous study contradicted the results of our study by reporting that fecal microbial diversity decreased from health to cirrhosis, while diversity increased from cirrhosis to early HCC (Ren et al., 2019). Their results may be attributed to the differences in regional 16S rRNA Miseq sequencing, and data analysis methods used in our study. In addition, our analysis also found that observed_ species, PD_ whole_ Tree, ACE indices, which mainly represent the sample sequencing depth of α-diversity, were positively correlated with count of CD3^+^, CD4^+^, and CD8^+^T cells (Song et al., 2022). This indicates that sufficient sample size and depth analysis should be ensured when observing the association between the microbiota and tumor immune effects. This study also observed that the composition of intestinal microbiota in LC patients changes significantly prior to HCC compared with healthy people, which is similar to the phenotypic changes associated with the tumor immune response. For example, our previous research found that the level of PD-1+, TIGIT+, TIM-3 co-inhibitory molecules of adaptive immune CD4+, CD8+T, NK cells representing the exhaustion function gradually increased during the pathological process of chronic hepatitis B virus to cirrhosis and progressed HBV-HCC (Liu et al., 2019; Yu et al., 2021). An increased ratio of *Bacterioid* to *Firmicutes* (B/F) has been reported to be associated with tumor immunity. The abundance of *Firmicutes* and *Bacteroidetes* in CHB, LC and HCC patients was lower than healthy control. In addition, *Proteobacteria* was significantly more abundant in LC and HCC. We define this phenomenon as “pre-dysfunction”. Thus, changes in gut microbiota may exert potential pathogenic effects on HBV-HCC as well as on tumor immunity.

It is reported that *Lactobacillus* and other levels of bacteria may significantly inhibit the inflammatory response of tumors. It belongs to a beneficial flora and significantly reduces in HCC (Dou et al., 2021; Hao et al., 2021). An important result of our study is that *c: Bacilli, o: Lactobacillales*, *and f: Lactobacillus* were significantly increased in the HBV-HCC patients. This is inconsistent with previous reports. Then, we further strengthened the clinical treatments for HCC patients by extending the time period allowed for the us flora regulating drugs to 2 months. We analyzed the abundance of *Lactobacillus* and other level bacteria in HCC patients and generated the same result. Furthermore, we observed that the abundance of *Lactobacillus* in patients subjected to long-term bacterial regulation drug treatment was not consistent. We surmised that this may be attributed to the local inflammatory reaction to the tumor in HCC patients being more intense. The structure of gut microbiota is more complex and protective mechanisms of the body may induce abnormally high expression of *Lactobacillus via* negative feedback, causing it to be transported to the tumor microenvironment *via* the gut-liver axis to cope with the inflammatory reaction caused by tumor cells (Yang et al., 2022). In addition, the high level of lactic acid in the tumor microenvironment provides energy to tumor cells through the Warburg effect and aggravates the hypoxic environment, making it unsuitable for the survival and function of effector T cells and attenuating the anti-tumor immune function. An appropriate amount of *Lactobacillus*, which is known for its an anti-tumor role, indicating that its excessive production and transport *via* the gut-liver axis may be responsible for the immunosuppression in the local tumor microenvironment (Watson et al., 2021; Kumagai et al., 2022).

According to the report, fecal SCFA concentration is closely related to the incidence of colorectal cancer. Gut microbial metabolite butyrate can directly modulate antitumor CD8+ T cell response and improve the chemotherapy efficacy through ID2-dependent IL-12 signaling, suggesting that manipulation of gut microbial metabolites could be effective as a part of cancer therapy (He et al., 2021). Besides, they can not only promote the differentiation and function of anti-inflammatory macrophages, Treg cells, CD4+, CD8+cells, IL-10+IL-35+B cells but also IL-22-producing innate lymphoid cells 3 (ILC3), which are involved in maintaining the gut mucosal homeostasis (Su et al., 2022; Tilg et al., 2022). It provided strong evidence indicating that intestinal microorganisms may play a leading role in the tumor immune effect of chronic liver disease. Our research shows that HBV-CLD patients were able to detect a new core microbiome signature characterized by a significant increase in pro-inflammatory bacteria, such as *gamma-Proteus, Proteus, Klebsiella, streptococcus* as well as a reduction in the butyric acid producing bacterial with *Ruminococcus, oscillatory bacillus, clostridium ruptured* compared to the healthy people. This finding was consistent with those of previous studies (Huang et al., 2020; Kawanabe-Matsuda et al., 2022). We further found that HBV-HCC patients displayed more significant immunosuppression. These have negative correlation with the lower butyrate producing bacteria. This study revealed the potential shown by fecal microorganisms for regulating the anti-tumor effect of HBV-HCC.

The ability of microbiota and its metabolites to regulate immune response is associated with better prognoses in HCC. The increase in circulating Tregs in the peripheral blood of HCC patients has been proved to be a major factor in predicting poor prognoses (Bachem et al., 2019; He et al., 2021). In addition, the metabolites of lactic acid may stably exert continuous immunosuppressive effects on tumor infiltrating Treg cells (Kumagai et al., 2022). Recent studies have confirmed the importance of intestinal microbiota in promoting the anti-tumor killing function of CD8+T cells (Luu et al., 2018; Trompette et al., 2018). Considered together, the results of our studies as well as those of others, indicate that HBV-CLD patients, in general, and HCC patients, in particular, may benefit from treatment plans involving gut-related intervention strategies that reactivate the patient’s anti-tumor response.

Our study was affected by some limitations. Firstly, individual differences especially dietary habits, age, and gender factors are variable and exhibit deviations. Therefore, the larger the sample size used, the smaller the difference between groups, the more stable the results. However, due to the effects exerted by the current COVID-19 epidemic on our hospital, some difficulties were encountered when recruiting samples for the study. In addition, this cross‐sectional study does not reveal whether microbiota exerts a direct impact on disease progression. Finally, this study concentrated on phylum, family, genus levels and did not conduct an in-depth investigation at the species or deeper levels. However, we did use these samples to conduct metabonomic sequencing research to supplement these limitations.

## 5 Conclusions

In summary, we determined that HBV-HCC patients have specific gut microbiota disorders and more intense anti-tumor immunosuppression phenotypes compared with healthy individuals and HBV-LC. T cell immunosuppression in the tumor microenvironment is correlated with an increase in inflammatory and lessen in butyric acid producing gut microbiota. It may provide a most effective shortcut to targets and help reverse the anti-tumor immune response of HBV-HCC patients.

## Data availability statement

The original contributions presented in the study are included in the article/**Supplementary Material**. Further inquiries can be directed to the corresponding author.

## Ethics statement

The study was approved by the ethics committee of Beijing Ditan Hospital, Capital Medical University and was conducted in accordance with the standards of the Declaration of Helsinki. The patients/participants provided their written informed consent to participate in this study.

## Author contributions

XW designed the study. FY, QZ, KS, YZ, BZ, and YB: sample collection and data acquisition. FY: statistical analysis, interpretation of data, and manuscript writing. All authors contributed to the article and approved the submitted version.
