# Supplementary figures and images for: Gut microbiota dysbiosis with hepatitis B virus liver disease and association with immune response
Source: Front Cell Infect Microbiol. 2023 May 2;13:1152987. doi: 10.3389/fcimb.2023.1152987 (PMC10185817; doi:10.3389/fcimb.2023.1152987)

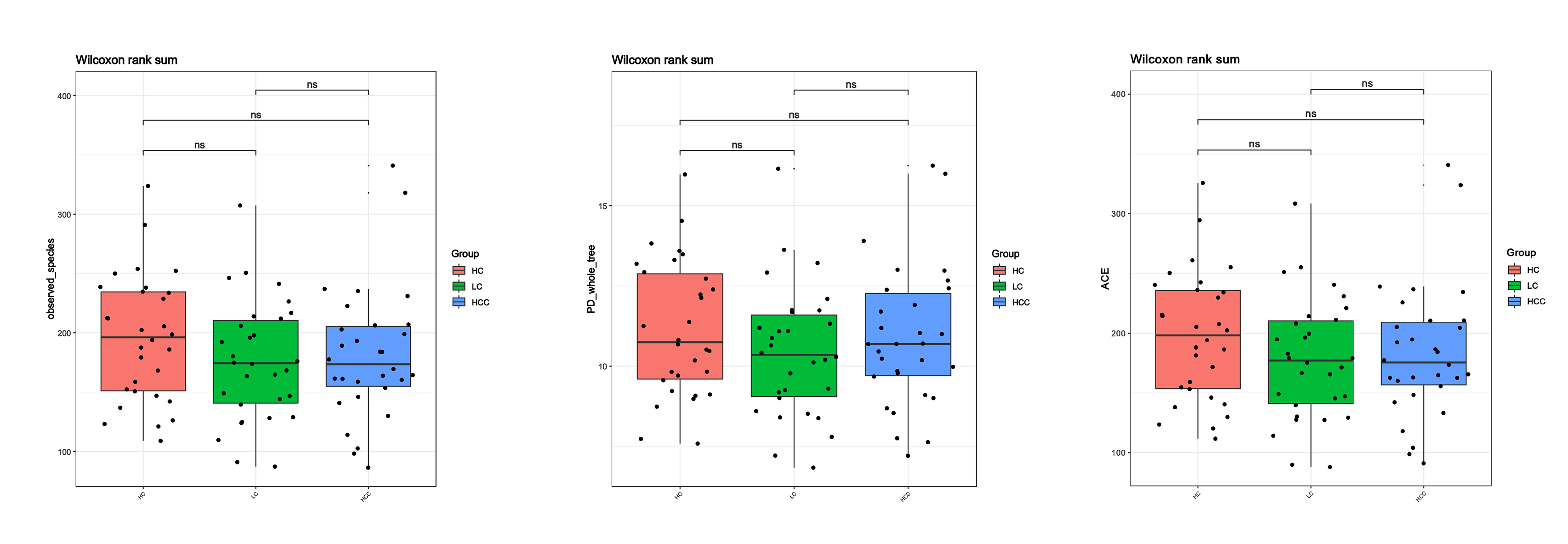

Supplement: Supplementary file 1 [file Image_1.tif]

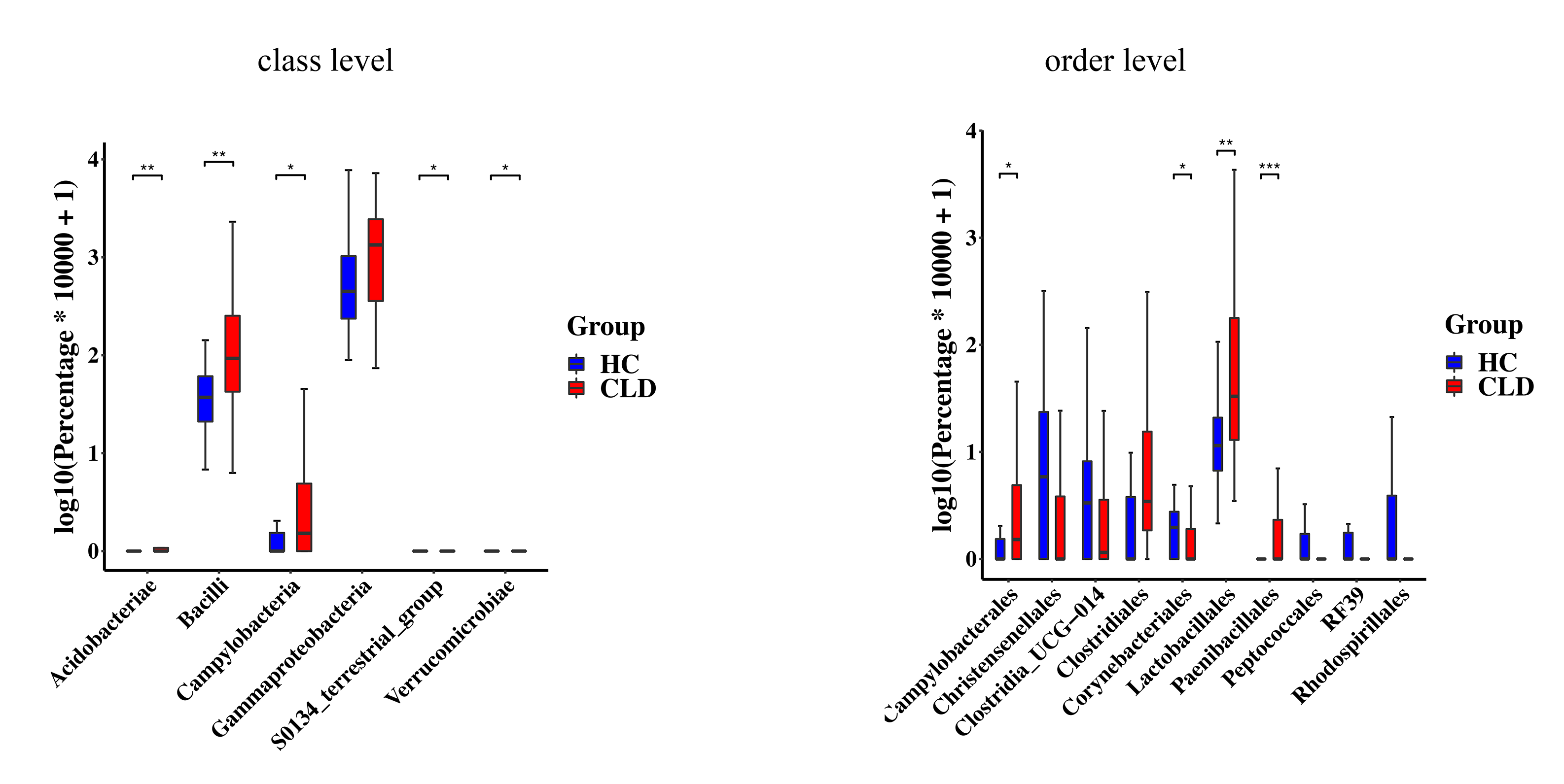

Supplement: Supplementary file 2 [file Image_2.tif]
